# Allogeneic Transplantation for Chronic Lymphocytic Leukemia

**DOI:** 10.4084/MJHID.2010.026

**Published:** 2010-09-07

**Authors:** Luca Laurenti, Michela Tarnani, Patrizia Chiusolo, Federica Sorà, Simona Sica

**Affiliations:** Istituto di Ematologia, Policlinico “A. Gemelli”, Universita’ Cattolica del Sacro Cuore, Rome, Italy

## Abstract

Even if Chronic lymphocytic leukemia (CLL) often has an indolent behavior with good responsiveness to cytoreductive treatment, about 20% of the patients, so called “poor-risk” patients, show an aggressive course and die within a few years despite early intensive therapies. Criteria for poor-risk disease according to the European Bone Marrow Transplantation (EBMT) CLL Transplant Consensus are: purine analogue refractoriness, early relapse after purine analogue combination therapy, CLL with p53 lesion requiring treatment.

Allogeneic transplant has potential curative role in CLL, however burden with very high transplant related mortality (TRM) rates of 38–50%. A major advance in reducing the short-term morbidity and mortality of allogeneic stem cell transplantation (SCT) has been the introduction of non-myeloablative or reduced intensity conditioning (RIC) regimens to allow engraftment of allogeneic stem cells. There is no doubt that the crucial therapeutic principle of allo-SCT in CLL is graft versus leukemia (GVL) activity.

The major complications of allogeneic SCT in CLL are: chronic graft-versus-host-disease (GVHD) affecting quality of life, high graft rejection and infection rates correlated with preexisting immunosuppression. Disease relapse remains the major cause of failure after RIC allo-HCT in CLL patients.

Sensitive minimal residual disease (MRD) quantification has strong prognostic impact after transplant.

## Introducion:

Chronic lymphocytic leukemia (CLL) is the most common adult leukaemia in the Western World^1^ and has long been considered an indolent disease affecting elderly patients, with a median age at diagnosis of approximately 72 years.

In parallel with improvements in treatment outcome, recent advances in the understanding of CLL biology have allowed us to identify selected subgroups of patients with adverse characteristics such as chromosomal abnormalities,^2^ immunoglobulin heavy chain (IgVh) gene mutational status,^3^ zeta associated protein 70 (ZAP70)^4^ and CD38 expression,^5^ some of whom have projected survival as short as 3 years.

Even if CLL often has an indolent behavior with good responsiveness to cytoreductive treatment, about 20% of the patients who need treatment show an aggressive course and die within a few years of diagnosis despite early institution of intensive therapies. These so called “poor-risk” patients are characterized by preexisting or rapidly developing resistance to conventional chemotherapy, including modern purine analogue-antibody combination regimens^6^,^7^ with a dismal survival of less than 12 months.^8^,^9^

Although alemtuzumab, lenalidomide, and flavopiridol, show promising activity in some patients with poor-risk CLL, none of these compounds seem to be able to change the natural course of poor-risk CLL.^10^–^14^

## Transplant in CLL:

Myeloablative allogeneic SCT has several theoretical advantages over autologous SCT. Firstly, any potential tumor contamination of the stem cell product is eliminated even if new data are emerging about the potential possibility of CLL transmission by unrecognized CLL/MBL (Monoclonal B Lymphocytosis) donor.^15^ Secondly, there is the potential for the graft versus leukaemia (GVL) effect to eliminate chemotherapy-resistant leukaemia cells by immune mechanisms. Moreover, in contradistinction with the results of autologous SCT plateaus in the survival curves emerge 1–2 years following allogeneic transplantation.

The enthusiasm for myeloablative allogeneic SCT in CLL, using either related or unrelated donors, is however tempered by registry reports showing very high TRM rates of 38–50%: these published data showed that approximately two-thirds of allotransplanted CLL patients will succumb either to TRM or to recurrent disease, and approximately one-third will be cured of their disease.^16^,^17^ Moreover patients with chemosensitive disease have significantly better outcomes than patients with refractory disease, suggesting that an earlier application of allogeneic SCT may further improve transplantation outcomes.

A major advance in reducing the short-term morbidity and mortality of allogeneic SCT has been the introduction of non-myeloablative or reduced intensity conditioning (RIC) regimens to allow engraftment of allogeneic stem cells. So the RIC regimens were developed in the 1990s to allow allo-SCT in older patients or younger patients with comorbidities and the total number of allogeneic transplantations for CLL patients in Europe, has almost treble in the last 7 years. In the last decade, many reports, which enrolled primarily patients with chemorefractory end-stage disease, have stressed the potential curative role of RIC allo-SCT in CLL.

There is no doubt that the crucial therapeutic principle of allo-SCT in CLL is GVL activity. This evidence derives from some interesting observations such as: decreasing relapse incidence over time even in RIC alloSCT, in contrast to autologous SCT or other intensive therapies,^16^–^22^ achievement of durable clinical and molecular responses due to antitumor activity, [23] reduced relapse rates in patients with chronic graft-versus-host disease (GVHD),^20^,^24^ increased relapse rates associated with T cell–depleted grafts^25^,^26^ and efficacy of donor lymphocyte infusions (DLIs) in the post-transplant relapse.^25^,^19^,^27^

Multiple single-center phase 2 studies have evaluated several different RIC regimens with different myeloablative and immunoablative potential (**Table 1**).

Ritgen et al demonstrated that in the majority of cases achievement of minimal residual disease (MRD) negativity was clearly linked to immune intervention, such as tapering of immunosuppression or donor lymphocyte infusions that lead to develop chronic GVHD, also he suggests that a “GVL escape” mechanism might be acquired during the post-transplantation course probably caused by survival of clonogenic cells at “GVL sanctuary” sites.^23^ Farina et al confirmed GVL activity in patients in clinical complete response (CR) after RIC alloSCT showing delayed clearance or decrease of MRD levels upon chronic GVHD in clinical CR.^24^ Both studies also detected that MRD negativity 1 year post transplant was durable over the entire follow-up period in more than 90% of patients and predictive for the virtual absence of clinical relapse. In conclusion, MRD kinetics studies consistently indicate that permanent MRD negativity after alloSCT for CLL can be reached in the context of chronic GVHD and/or immunomodulating intervention. Both the durability of MRD remission and its sensitivity to immunomodulation strongly suggest that GVL is effective in CLL.

Ex-vivo and in-vivo T-cell depletion are effective means of preventing acute and chronic GVHD after alloSCT. In CLL, however, T-cell depletion has been associated with increased relapse and rejection rates.^26^–^28^ Now-a-day, considering the scanty data, the role of this type of transplantation in CLL is controversial.

## “Poor risk” patients: indications and Allo-SCT timing in CLL:

European Bone Marrow Transplant (EBMT) guidelines have now been established outlining indications for SCT in CLL^29^ (**Table 1**). In the absence of prospective randomized trials, the Consensus recommendations were based on evidence of grade II or less.

Criteria for poor-risk disease according to the EBMT CLL Transplant Consensus are: purine analogue refractoriness, early relapse after purine analogue combination therapy, and CLL with p53 lesion requiring treatment.

However considering the recent improvements in CLL treatment outcome, now-a-day one should test other innovative and very promising therapeutically approach before giving the indication to alloSCT, such as: antibody-purine analogue combination regimens as first-line or salvage treatment,^30^,^31^ combined alemtuzumab therapies useful in fludarabine refractory and 17p-/p53 patients as showed by the German CLL Study Group (GCLLSG) CLL2H study,^12^ and novel agent flavopiridol and lenalidomide capable to achieve 30% to 40% response rate when used as salvage therapy in fludarabine-refractory or del 17p-CLL.^13^,^14^ Moreover these approaches may be helpful to achieve remission, a basic prerequisite for successful alloSCT.^32^

The EBMT guidelines conclude that there is evidence-based efficacy of allogeneic SCT in CLL and that this procedure is indicated in high-risk CLL patients. It should be stressed that none of the EBMT categories requires assessment of biologic risk factors except for cytogenetics for detection of p53 deletions, who deserves allogeneic SCT in first response. Ongoing prospective clinical studies will determine the impact of biomarkers including IgVh mutational status and other cytogenetic abnormalities in identification of patients at sufficiently high risk to deserve use of allogeneic SCT in first CR.

Another crucial point in the allogeneic transplant is a better selection of patient, as recently suggested by Sorror.^21^ The FHCRC analysis revealed that comorbidities were more important than CLL-related variables for predicting progression free survival (PFS) and overall survival (OS).^21^ Another risk factor for non-relapse-mortality (NRM), usually correlated with the presence of comorbidities, is refractory disease at transplantation.^22^ Thus, it would appear prudent to limit allo-SCT to fit patients without evidence of refractory disease.

In conclusion, allo-SCT is the only therapy with curative potential in CLL and, in contrast to conventional treatment, with potential of providing long-term disease control even in patients with a very unfavorable biological and clinical risk profile (**Table 2**). However in addition to the disease risk, patient-related risk factors, such as age and comorbidity, have to be considered when the decision about allo-SCT is made.^21^,^33^

The larger RIC prospective trials uniformly show that in CLL the results of alloSCT are considerably impaired if the disease is not in remission at the time of transplantation, due to nodal bulks and/or chemotherapy resistance. Thus, alloSCT should be performed before CLL has advanced to a status of complete refractoriness or large resistant tumor burden in order not to miss the “window of opportunity” for a successful outcome. In eligible patients, alloSCT should be considered as soon as the first and/or the third EBMT criterion is met; on the contrary, the optimum timing for alloSCT in patients with early relapse (within 2 years) after purine analogue combination therapy (second EBMT criterion) needs to be defined by systematic studies.

On the other hand, RIC allo-HCT is able to mitigate the adverse prognosis conferred by purine analog resistance and unfavorable genetics. The EBMT guidelines identify a group of patients in whom available therapies are unlikely to achieve a prolonged disease-free survival.

## The choice of Conditioning regimen and stem cell source:

Several prospective RIC studies with a combined number of more than 300 patients included on the basis of modern CLL-specific risk stratification give relatively concordant results. In contrast, the experience with myeloablative conditioning in CLL largely relies on registry analyses and a few small single-center studies.^15^–^18^,^34^,^28^ Therefore, RIC rather than myeloablative conditioning represents the standard procedure for alloSCT in CLL.

The choice of RIC alloSCT as the standard procedure rather than myeloablative conditioning emerged by the fact that the crucial therapeutic principle of allotransplantation in CLL is GVL activity. That evidence for superior direct cytotoxic activity of myeloablative conditioning over RIC is lacking, and also one should consider that CLL is an elderly disease.^35^ At the same time an increased incidence of relapse after RIC in comparison with myeloablative conditioning was observed in a registry analysis, thus impaired disease control by RIC cannot be clearly ruled out.^20^ Thus, according to the individual situation, the optimum choice of conditioning regimens may vary: in the presence of comorbidity and sensitive disease reduced-intensity regimens appear to be more appropriate, high-intensity regimens might be preferable in younger patients with good performance status but poorly controlled disease.^36^,^29^

A matched sibling donor should be preferred for alloSCT in CLL whenever possible, but a matched unrelated donor (MUD) is a reasonable alternative if an HLA-identical sibling is not available. In fact from published RIC studies in CLL, unfavorable effect of MUD respect to sibling donor did not significantly emerge.^19^,^21^–^22^,^27^,^37^–^38^

Due to the median age of patients, experience with haploidentical transplant and cord blood transplant is very scanty in CLL, and it is unlikely that disease-specific evidence for benefit of these procedures can ever be obtained.

RIC allo-SCT was associated with a NRM of 11%–34%, a PFS of 34%–67%, and OS of 48%–72% depending on the conditioning regimen and follow-up. Long-term disease control was obtained in a substantial proportion of patients, particularly in those with chemosensitive and non-bulky disease at the time of transplantation.

In the CLL3X trial of the German CLL Study Group (GCLLSG) and also in Schetelig data, poor risk CLL as defined by purine-analogue refractoriness or presence of deletion 17p- had a similar outcome to patients without poor-risk characteristics in terms of PFS, with a median follow up of 2 years.^19^

The outcome of 82 patients treated at the FHCRC consortium using a non-ablative conditioning regimen (fludarabine and low-dose total body irradiation) was recently updated.^20^ Eighty-seven percent of patients had fludarabine refractory disease and 9% had del(17p).

At 5 years after RIC allo transplant, the NRM and relapse rate were 23% and 38% respectively, which translated into a OS of 50% and PFS of 39%. Patients with bulky lymphadenopathy (>5 cm) at the time of transplantation had a particularly poor outcome, with a 5-year relapse rate and PFS of 71% and 8% respectively. Extensive chronic GVHD was diagnosed in 50% of patients, but it resolved in most of them.^21^

A retrospective analysis of the EBMT on alloSCT in 44 patients with del 17p- CLL showed a 3-year PFS of 37% with no event occurring later than 3.5 years after transplant.^26^

We recently reported in a single centre experience the efficacy and feasibility of RIC allo-SCT in heavily pre-treated CLL patients, even if in a small number of patients and with a relatively short follow-up. In our series, 45% of patients were considered “high-risk” for fludarabine-refractory disease or 17p deletion. At median follow-up of 20 months (range 12-44) 80% of them are alive, confirming literature data.^39^

In conclusion, RIC alloSCT seems to be effective in poor-risk CLL, thereby overcoming the adverse prognostic impact of purine analogue refractoriness and del 17p-. However, active, unresponsive and bulky disease at the time of alloSCT still remains a predictor of an unfavorable outcome.^20^–^22^,^37^

## Complication of allogeneic SCT in CLL and post-transplant follow-up:

The major determinant of long-term morbidity affecting quality of life after alloSCT is chronic GVHD. Active chronic GVHD documented a significantly reduced long-term health status in patients allografted for various hematological malignancies.^40^ Sorror et al. reported a 5-year cumulative incidence of extensive chronic GVHD of 49% for related and 53% for unrelated recipients. However, in the majority of affected patients clinical symptoms of chronic GVHD resolved over time, allowing discontinuation of systemic therapeutic immunosuppression after a median of 25 months.^21^ Transplant-related long-term morbidity after alloSCT for CLL can be significant but is mainly restricted to those patients who have ongoing active chronic GVHD.

The high graft rejection rates remain a complication either of myeloablative or non-myeloablative transplant in CLL: it ranges between 18%– 20% in conventional conditioning,^16^,^41^ while in case of RIC regimen between 12–25% in T cell–depleted grafts and 5–6% in unmanipulated grafts.^21^,^38^,^42^ The possible explanations for this phenomenon could be the significant marrow infiltration in CLL patients at the time of transplantation, inversely correlated with outcome^37^,^42^ and the role played by host dendritic cells, which are seriously defective in CLL patients.^43^

Another complication is represented by the high infection rates correlated with preexisting immunosuppression. Infections are the cause of about 50% of all CLL-related deaths,^44^–^45^ primarily in fludarabine and/or alemtuzumab-refractory patients.^7^,^8^ The base of susceptibility to development infections is the immunosuppression before SCT both disease and therapy-related, rarely encountered in patients with other lymphoproliferative disorders, such as follicular lymphoma, whose main cause of death is disease progression.^46^ The multifactorial genesis should be secondary to hypogammaglobulinemia, defective T and natural killer cell function, neutropenia, and deficient complement activity.^47^ Moreover in recent reports the risk of infections has been clearly correlated with presence of GVHD^21^,^38^,^43^,^48^ and refractory disease.^8^,^49^

Disease relapse remains the major cause of failure after RIC allo-HCT in CLL patients. Early relapses are correlated with chemorefractory disease at the time of transplantation, perhaps due to the ineffectiveness of RIC regimens in controlling the disease before the graft vesus CLL (GVCLL) effect can take place.

Late relapse an be explaned with different mechanism including: CLL clonal evolution, the development of tolerance^25^ and the survival of tumor cells in “GVCLL sanctuary sites” based on the negative impact of bulky lymphadenopathy at the time of transplant on PFS,^21^ inadequate GVL effect to allow a complete disease eradication. It should be noted that a significant percentage of these late relapses occurred in lymph nodes in the absence of bone marrow or peripheral blood involvement, or even in patients with MRD negative status.^9^,^28^,^34^,^50^–^52^

Sensitive MRD quantification can be studied in CLL by polymerase chain reaction (PCR) or flow cytometry-based assays and has strong prognostic impact after transplant. The generally delayed decline of the MRD level and its close correlation with immune-relevant events strongly supports the assumption that GVL activity is the crucial contribution to tumor control in the allo-SCT. GVL-induced MRD negativity after allogeneic transplant is highly predictive of freedom from relapse.^24^

Moreover in CLL quantitative MRD monitoring seems to be a valid instrument for sensitive guidance of immune interventions aimed at disease eradication after allo-SCT. In contrast to donor’s lymphocyte infusion (DLI) upon clinical relapse which often shows only limited benefit in CLL,^19^,^33^ MRD-triggered pre-emptive DLI can be highly effective.^53^

The best approach to post transplant immunotherapy, including monoclonal antibody^38^ in CLL needs further study. Some of them, although a still short follow-up, show very promising results and the use of monoclonal antibody in the conditioning or just after transplant, could improve the results of allogeneic stem cell transplantation.^54^ Rituximab given concomitantly with RIC allo-SCT or DLI may facilitate disease control^43^ probably due, not only to direct cytotoxicity, but also to modulation of the GVCLL effect. In addition, maintenance therapy with monoclonal antibodies or immunomodulatory drugs with proven activity in poor-risk CLL, such as lenalidomide, could be explored in future trials to minimize the risk of relapse.

## Figures and Tables

**Table 1:** RIC allogeneic SCT for CLL

**n**	**Total patients**	**Age Year (range)**	**Prior Regimens (median)**	**Chemo-Refractory**	**Prior Auto-SCT**	**Donor**	**TRM**	**GVHD Acute (Grade II–IV)**	**GVHD Chronic (extensive)**	**Survival**	**Reference**
30	30	50 (12–63)	3	47%	n.d.	50% related 50% unrelat ed	13% (overall)	56%	21%	OS 72% 2 yr PFS 67%	Schetelig et al 2003
77	77	54 (30–66)	3	33%	10	81% related	18% (12m)	34%	58%	OS 72% 2 yr PFS 56%	Dreger et al 2003
46	46	53 (35–67)	5	57%	10	33% related 67% unrelated	17% (overall)	34%	43%	OS 54% 2 yr PFS 34%	Brown et al 2006
41	41	54 (37–67)	3	27%	11	58% related 42% unrelated	5% (100 d)	10% (III–IV)	33%	OS 51 2 yr PFS 45%	Delgado et al 2006
39	39	57 (34–70)	3	n.d.	n.d.	90% related 10% unrelated	26% (overall)	45%	58%	OS 48% 4 yr PFS 44%	Khouri et al 2006
82	82	82 (42–72)	4	87%	4	63% related 37% unrelated	25% (overall)	55%	49% related 53% unrelated	OS 50% 5 yr PFS 45%	Sorror et al 2008
11	11	57 (49–70)	4	45%	3	73% related 27% unrelated	27% (overall)	18%	36%	OS 73% 2,5 yr PFS 55%	Laurenti et al 2010
25	25	57 (45–70)	3	n.d.	6	88% related 12% unrelated	13% (overall)	40% (I–IV)	60% (overall)	OS 55% 5 ys PFS 43%	Iori et al 2010 (abs)

**Table 2:** Criteria for poor-risk disease according to the EBMT CLL Transplant Consensus.

**Allo-SCT is a reasonable treatment option in poor-risk CLL including:**
– Fludarabine resistance - non-response or early relapse (< 12 months) after purine analogue-based therapy – Relapse < 24 months after purine analogue combinations or auto-SCT (+ high-risk genetics) – p53 mutation with treatment indication

**Auto-SCT indicated in clinical trial only.**
